# Editorial: Insights into Parkinson's disease and aging related movement disorders

**DOI:** 10.3389/fnagi.2023.1193197

**Published:** 2023-05-17

**Authors:** Robert B. Petersen, Benjamin Walter

**Affiliations:** ^1^College of Medicine, Central Michigan University, Mt. Pleasant, MI, United States; ^2^Cleveland Clinic, Cleveland, OH, United States

**Keywords:** Parkinson's disease, ALS, microbiome, mitochondria, sleep

As suggested by the title of this Research Topic, this is a broad and diverse group of articles encompassing themes that expand beyond movement disorders into subjects pertinent to neurodegenerative diseases generally. As the second most common neurodegenerative disease, Parkinson's disease is especially important. One of the major challenges in the treatment of neurodegenerative disease is its early diagnosis. There are several challenges surrounding the early diagnosis of Parkinson's disease (Tolosa et al., 2021). An important and underappreciated class of diagnostic criteria is provided by the non-motor symptoms (e.g., constipation, urinary dysfunction, hyposmia) that may be some of the earliest presenting symptoms. The greatest opportunity for successful treatment is during the prodromal stage, underlining the importance of early diagnosis. In addition, there is still a dearth of information about the origins and targets for treating movement disorders. This Research Topic addresses some recent advances including risk factors, novel therapies and targets, assessment of disease progression, and association with other diseases.

As is the case with neurodegenerative disease generally, identifying modifiable risk factors and markers of disease progression is essential. Sleep has emerged as an important factor in neurodegeneration (Musiek and Holtzman, 2016). The original research article by Yi et al. explored the effect of sleep on the progression of Parkinson's disease, addressing both motor and non-motor symptoms. Both categories were found to be negatively impacted by poor sleep hygiene. A separate study by Zhou et al. examined oculomotor function and found that it is an early indicator of Parkinson's disease and can be used to monitor disease progression. An important observation provided by Kang et al. revolves around the association of gait disturbance and cognitive impairment. The results of this study emphasize the importance of evaluating specific aspects of gait disturbance in Parkinson's patients with mild cognitive impairment. Finally, Liu et al. investigated task-generated subjective fatigue in Parkinson's patients associated with regional blood flow changes; fatigue, while one of the most common non-motor symptoms, is underdiagnosed and may in fact be one of the earliest symptoms.

Targeting and treating movement disorders are also crucial efforts. Mitochondria have been implicated in the development of neurodegenerative diseases, and this is true of movement disorders as well. Silvian presented a mini-review on targeting the PINK/Parkin pathway in mitochondrial quality control as a potential treatment for Parkinson's disease. Chen et al. looked specifically at medium spiny neurons and found that a deficiency of Pitx3 resulted in many of the pathological features of Parkinson's disease, suggesting that enhancing Pitx3 may prevent disease and provide a marker for disease diagnosis. Chiu et al. addressed the increased subjective pain sensitivity associated with Parkinson's disease in a rat model. Using fetal tissue allografts, they showed that rats exhibited increased nociceptive thresholds and improved motor function. This study also showed the promise of using fMRI to analyze efficacy. Another major problem associated with movement disorders is fall risk, which can lead to a variety of sequelae including hip fracture. Oddsson et al. demonstrated that a prosthesis provided significant improvement in gait and balance during extended use and suggested that the long-term effect indicates that this is not simply a placebo effect.

While it is well-known that agricultural work is a risk factor for PD, Zhang et al. provided an extensive epidemiological analysis in China that suggests that farmers are at increased risk for ALS. This provides a basis for searching for broader links between pesticide exposure and neurodegenerative conditions beyond PD.

The importance of the gut microbiome to brain health/function is increasingly apparent. In their review, Zeng et al. examined the hypothesis that Parkinson's disease may be derived from the gut, with a focus on whether gastrointestinal diseases are a factor. An interesting observation is that Parkinson's disease and gastrointestinal diseases may have bi-directional correlations, i.e., Parkinson's may predispose to gastrointestinal disease, and gastrointestinal disease may predispose to Parkinson's disease.

It is hoped that this diverse collection of articles will provide focus for future research into Parkinson's disease and age-related movement disorders. Thanks are extended to the authors, editors, and reviewers for helping to assemble and clarify this diverse collection of contributions.

## Author contributions

All authors listed have made a substantial, direct, and intellectual contribution to the work and approved it for publication.
